# Hypoxic Signaling Pathways in Carotid Body Tumors

**DOI:** 10.3390/cancers16030584

**Published:** 2024-01-30

**Authors:** Kangxi Cao, Wanzhong Yuan, Chaofan Hou, Zhongzheng Wang, Jiazhi Yu, Tao Wang

**Affiliations:** Department of Neurosurgery, Peking University Third Hospital, Beijing 100191, China; caokangxi@hsc.pku.edu.cn (K.C.); ywz@stu.pku.edu.cn (W.Y.); 2111210370@stu.pku.edu.cn (C.H.); 2211210432@pku.edu.cn (Z.W.); pkuhscyujiazhi@pku.edu.cn (J.Y.)

**Keywords:** carotid body tumor, hypoxia, SDH, HIF, VEGF, inflammatory cytokines

## Abstract

**Simple Summary:**

Carotid body tumors (CBTs) are rare tumors and only appear in 1–2 individuals per 100,000. The etiology of CBTs remains unclear; however, SDH mutations and familial inheritance have been reported to be related to CBTs. SDH complexes play crucial roles in aerobic respiration, and SDH mutations in CBTs have been reported to be associated with hypoxia. Hypoxic signaling pathways, specifically hypoxic markers, have attracted more research attention in tumor exploration. However, the existing literature on these signaling and markers lacks a systematic review. Also, therapeutic approaches in CBTs based on hypoxic signaling are rarely used in clinics. In this review, we highlighted the role of hypoxic signaling pathways and markers and their potential implications in the initiation and progression of CBTs. Our findings underscore the involvement of the SDH family, the HIF family, VEGFs, and inflammatory cytokines in tumorigenesis and treatment based on them. Moreover, this review offers valuable insights for future research directions on understanding the relationship between hypoxia and CBTs.

**Abstract:**

Carotid body tumors (CBTs) are rare tumors with a 1–2 incidence per 100,000 individuals. CBTs may initially present without apparent symptoms, and symptoms begin to arise since tumors grow bigger to compress surrounding tissue, such as recurrent laryngeal nerve and esophagus. Also, the etiology of CBTs remains unclear since it is more likely to occur in those who live in high-altitude areas or suffer from chronic hypoxic diseases such as COPD. SDH mutations and familial inheritance have been reported to be related to CBTs. SDH complexes play crucial roles in aerobic respiration, and SDH mutations in CBTs have been reported to be associated with hypoxia. Hypoxic signaling pathways, specifically hypoxic markers, have attracted more research attention in tumor exploration. However, the existing literature on these signaling and markers lacks a systematic review. Also, therapeutic approaches in CBTs based on hypoxic signaling are rarely used in clinics. In this review, we concluded the role of hypoxic signaling and markers and their potential implications in the initiation and progression of CBTs. Our findings underscore the involvement of the SDH family, the HIF family, VEGFs, and inflammatory cytokines (ICs) in tumorigenesis and treatment. Of particular interest is the role played by SDHx, which has recently been linked to oxygen sensing through mutations leading to hereditary CBTs. Among the SDH family, SDHB and SDHD exhibit remarkable characteristics associated with metastasis and multiple tumors. Besides SDH mutations in CBTs, the HIF family also plays crucial roles in CBTs via hypoxic signaling pathways. The HIF family regulates angiogenesis during mammalian development and tumor growth by gene expression in CBTs. HIF1α could induce the transcription of pyruvate dehydrogenase kinase 1 (PDK1) to inhibit pyruvate dehydrogenase kinase (PDH) by inhibiting the TCA cycle. Then, carotid body cells begin to hyperplasia and hypertrophy. At the same time, EPAS1 mutation, an activating mutation, could decrease the degradation of HIF2α and result in Pacak–Zhuang syndrome, which could result in paraganglioma. HIFs can also activate VEGF expression, and VEGFs act on Flk-1 to control the hyperplasia of type I cells and promote neovascularization. ICs also play a pivotal signaling role within the CB, as their expression is induced under hypoxic conditions to stimulate CB hyperplasia, ultimately leading to CBTs detecting hypoxic areas in tumors, and improving the hypoxic condition could enhance photon radiotherapy efficacy. Moreover, this review offers valuable insights for future research directions on understanding the relationship between hypoxic signaling pathways and CBTs.

## 1. Introduction

**Figure 1:**
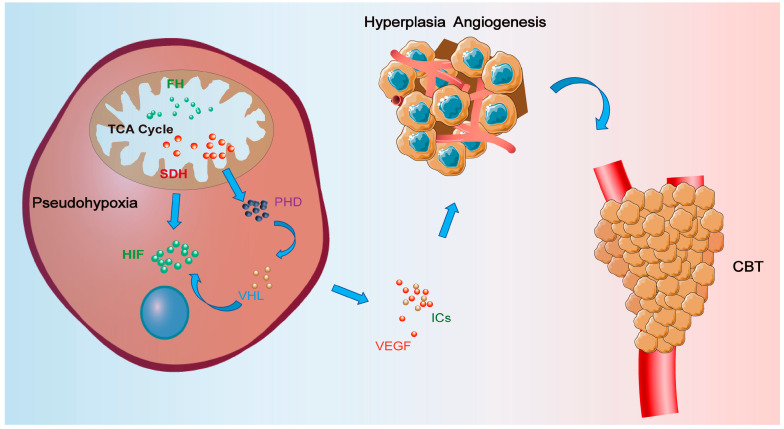
The hypoxic signaling pathways in CBTs. SDH and FH participate in the TCA cycle in mitochondria. Any mutations of SDH or FH can inhibit PHDs and result in the inappropriate elevation of HIF activity at any given level of PO_2_, also called pseudohypoxia. The hypoxia condition can increase the expression of VEGFs and ICs, resulting in angiogenesis in carotid body and the hyperplasia of carotid body cells. The former processes may finally result in CBTs.

Carotid body tumors (CBTs), or carotid glomus tumors, are rare neuroendocrine neoplasms with an estimated incidence of 1–2 per 100,000 individuals and constitute approximately 0.5% of head and neck tumor cases [1,2,3,4]. CBTs, a type of paraganglioma, are situated posterior to the bifurcation of the common carotid artery and present as a slow-growing, non-functional, and pulsatile cervical mass, usually described as an incidental finding in middle-aged females [4,5,6]. As these tumors grow and compress adjacent tissues and organs, patients may develop symptoms such as dysphonia, dysphagia, and Horner’s syndrome. The precise pathogenesis of CBTs remains elusive, with hypoxic signaling being a well-established contributing factor [7]. Chronic hypoxic conditions such as COPD or prolonged exposure to high altitudes can increase the burden on the carotid body chemoreceptor cells to compensate for reduced PO_2_ levels [8]. The glomus cells of the carotid body depolarize within milliseconds in response to hypoxemia using incompletely understood mechanisms due to oxygen sensing [9]. In hypoxic conditions, the transcription of messenger RNAs (mRNAs) under the control of HIFs is decreased in carotid body cells, inducing a series of hypoxic signaling and possibly resulting in CBTs. To overcome hypoxic conditions, mammals developed fundamental adaptive mechanisms for hypoxia, including increased ventilation and cardiac output, enhanced blood vessel growth, and circulating red blood cell numbers. At the cellular level, ATP-consuming reactions are suppressed, and metabolism is altered until oxygen homeostasis is restored. During these processes, the SDHx, HIFs, VEGFs, and inflammatory cytokines play crucial roles in the occurrence of CBTs; the relations of these factors and CBTs are shown in Figure 1, and this review shows the progress of hypoxic signaling research in CBTs and its potential pathological process based on Figure 1.

## 2. Anatomic and Physiologic Basis of Hypoxic Signaling in the Carotid Body (CB)

The carotid bodies house the peripheral chemoreceptors, essential components of the ventilatory control system that regulates the chemical composition of arterial blood [10]. In 1743, Albrecht Von Haller first described the anatomy of the carotid body. Carotid bodies are strategically located at the bifurcation of the common carotid artery, which supplies blood to the brain. These specialized structures respond to changes in oxygen, carbon dioxide, and metabolic acidosis, triggering rapid respiratory responses to optimize oxygen delivery and facilitate carbon dioxide elimination. In addition, CBs can detect low glucose levels, temperature fluctuations, and changes in osmolarity. Evidence suggests that they play a role in regulating airway resistance and cerebral blood flow [11].

In 1953, Gray et al. found that well-oxygenated tumor cells responded quicker to radiotherapy than hypoxic cells [12]. Tumor hypoxia was first proposed in 1955 by Thomlinson et al. in a study on the tumor tissues of patients with lung cancer [13], and, following 60 years of clinical and experimental research, scientists have confirmed that the hypoxic state is a widespread trait in a variety of solid tumors. The CBs comprise type I and type II cells, with the former involved in O_2_ sensing while the latter serves as glia-like sustentacular cells [14]. These cells are organized into clusters characterized by a central core of type I cells surrounded by a shell of type II cells. However, the complete mechanisms underlying O_2_ sensing remain poorly elucidated. In the study of Crapo et al. [15], changes in the partial pressure of oxygen in the arteries (PaO_2_) in healthy people at sea level and 1400 m above sea level were described in detail. The average PaO_2_ of a non-smoking healthy person younger than 65 at sea level is 99.8 mmHg, while the average PaO_2_ at 1400 m above sea level is 79.2 mmHg. For those older than 65 years, PaO_2_ decreased from 88.7 mmHg to 70.8 mmHg. This significant change in PaO_2_ leads to hypertrophy and hyperplasia in the carotid body. This was also confirmed in the study of van den Berg, R. [16] which showed that the average weight of the carotid body at sea level was 20 mg, while the average weight of the carotid body at high altitude increased to 60 mg, and the incidence of carotid body tumors also increased by nearly ten times.

It is widely believed that membrane ion channels play a critical role in the process and that low oxygen levels inhibit K+ currents through the CB glomus cell membrane [17]. This leads to membrane depolarization which then triggers calcium ion influx and activates a complex cascade of events within the glomus cell [17]. The activity of oxygen-sensitive K+ channels may also be modulated by intracellular substances, such as reactive oxygen species and ATP, as well as organelles, including mitochondria and membrane-bound heme-containing protein complexes [18], necessitating further investigation.

## 3. Hypoxic Signaling and Related Functions in CBTs

### 3.1. The Succinate Dehydrogenase (SDH) Signaling Pathway

In 2000, Baysal et al. [19] used linkage analysis and positional cloning methods to report for the first time that the defective mutation of the *SDHD* gene existed in paraganglioma. Subsequent investigations revealed mutations in other mitochondrial *SDH* subunits, namely *SDHA*, *SDHB*, and *SDHC* genes. *SDHx*-mutated paragangliomas lack SDH functions and exhibit metabolic changes, including reductive glutamine carboxylation and increased pyruvate consumption, to replenish aspartate pools through pyruvate carboxylation [20,21]. These metabolic alterations are observed in response to the loss of SDH function and contribute to the adaptation of tumor cells to hypoxic conditions. Additionally, *SDHx*-mutated paragangliomas have lower ATP/ADP/AMP levels, indicating a disruption in energy metabolism [22,23]. The activities of respiratory chain complexes I, III, and IV are increased in *SDHx*-mutated tumors to partially compensate for the SDH or complex II loss. These changes in respiratory chain activities can increase reactive oxygen species (ROS) production, which may signal oxygen insufficiency to prolyl hydroxylases (PHDs) and contribute to activating pathways associated with pseudohypoxia [24,25]. *SDHx* and fumarate hydratase (*FH*) mutations could lead to the persistence of HIFα in normal oxygen conditions [26]. Then, variants in *SDH* genes lead to complex II dysfunction, elevated succinate levels, the inhibition of prolyl hydroxylase (which typically regulates *HIFα*, thereby leading to increased activity of HIFα), and the inhibition of DNA demethylases, leading to global tumor DNA hypermethylation [27].

The *FH* gene encodes for fumarate hydratase, a TCA cycle enzyme that catalyzes the step following the succinate dehydrogenase, allowing the hydration of fumarate to malate. FH was recently involved in PPGL development. Patients with *FH* mutation had metastatic or multiple PPGL in 40% of cases [28]. The inactivation rates of FH and another TCA cycle component, SDH, have both been associated with abnormalities of cellular metabolism, responsible for the activation of hypoxic gene response pathways and epigenetic alterations, such as DNA methylation [29].

SDH oxidizes succinate into fumarate with the donated electrons and then participates in the electron transport chain in the TCA cycle [30,31]. If any component of the mitochondrial complex (that is, SDHA, SDHB, SDHC, SDHD, SDHAF1, or SDHAF2) is lost, then the entire SDH complex either becomes unstable or does not form. This has been reported to be associated with CBTs [32], and the mutation rates of *SDHx* in CBTs in previous research are shown in Table 1. Carriers of *SDHA* variants, a flavoprotein in the mitochondrial matrix, may lead to energy metabolism dysfunction, resulting in conditions such as Leigh syndrome or exercise intolerance [33]. Although pathogenic variants of *SDHA* leading to CBTs are rare due to low penetrance, patients with *SDHA*-associated CBTs may have an increased risk of contracting metastatic disease [34,35,36]. Carriers of *SDHB* variants may develop pheochromocytoma, extra-adrenal paraganglioma, and occasionally CBTs, with a metastasis incidence of approximately 23–25%, the highest among all genes associated with paraganglioma [26,37]. SDHC is a membrane-anchoring protein containing one heme essential for ubiquinone binding. Carriers of *SDHC* variants are more frequently associated with pheochromocytoma and paraganglioma [38,39,40]. The *SDHD* gene, associated with hereditary CBTs when its function is lost due to mutations, has recently been suggested to be involved in oxygen sensing [26]. *SDHD* variant carriers may develop multifocal pheochromocytoma and extra-adrenal paraganglioma, with metastases occurring in approximately 8% of cases [26]. Piruat et al. [33] generated a *SDHD* knockout mouse, a mammalian model lacking a protein from the electron transport chain, and their experimental findings demonstrated that CB responsiveness to hypoxia remains intact in heterozygous *SDHD* +/− mice; however, the loss of an *SDHD* allele was found to result in the abnormal enhancement of resting CB activity. This overactivity is associated with subtle glomus cell hypertrophy and hyperplasia, indicating that the constitutive activation of *SDHD* +/− glomus cells precedes CB tumor transformation [33]. Gimenez-Roqueplo’s study demonstrated that *SDHD* gene mutation results in the complete loss of complex II activity in the mitochondrial respiratory chain and is linked to the stimulation of angiogenic factors, which may facilitate or trigger tumorigenesis in paraganglia tissues [41]. SDHAF2 functions as a mitochondrial assembly factor for SDHA, which is essential for the activity of the succinate dehydrogenase complex. Mutations in *SDHAF2* have been linked to paraganglioma [42].

### 3.2. The Hypoxia-Inducible Factor (HIF) Signaling Pathway

Besides the *SDH-FH-HIF* pathway discussed above, another HIF-related hypoxic pathway is the *PHD*-von Hippel–Lindau (*VHL*)-*HIF* pathway. The HIF subunits undergo degradation in the proteasome under normoxic conditions through a mechanism that involves active PHD enzymes and the subsequent interaction of HIFs with VHL proteins, a component of the protein complex possessing ubiquitin ligase E3 activity [45]. A missense mutation partially impairs the binding of the VHL protein to the hydroxylated HIF-1α subunits, resulting in an inappropriate elevation in HIF activity at any given level of PO_2_ [46]. Hypoxic conditions suppress the activities of PHD enzymes, resulting in the stabilization and functional activation of the HIF complex [46]. HIFs are transcription factors that orchestrate various adaptive responses to hypoxia and are critical regulators in maintaining oxygen homeostasis [47,48,49]. The *PHD-VHL-HIF* pathway implicated in the cellular response to hypoxia plays a pivotal role in tumor initiation and progression [50]. The central convergence point of oxygen-sensing pathways is represented by the hypoxia-inducible factors, HIF1α and HIF2α, which are encoded by the genes *HIF1A* and *EPAS1*, respectively, and their expressions in the carotid body are shown in Table 2 [51].

The transcription factor HIF1α and HIF1α-targeted genes play a pivotal role in the metabolic adaptation associated with both hypoxia and pseudohypoxia. They could participate in cellular processes, encompassing metabolic adaptation to oxygen and nutrient deprivation, angiogenesis, cell proliferation, apoptosis, adhesion, migration, and survival [52]. HIF1α can induce the transcription of pyruvate dehydrogenase kinase 1 (*PDK1*), an inhibitor of pyruvate dehydrogenase (PDH), resulting in the inhibition of the TCA cycle when carotid body cells are exposed to hypoxic conditions [53]. In this manner, HIF1α coordinates the metabolic adaptations that enable cells to acclimate to hypoxia [52]. CB cells undergo hyperplasia and hypertrophy to cope with hypoxia, particularly the type I CB cells [54], which may serve as a potential mechanism underlying CBTs.

Unlike HIF1α, HIF2α exhibits more restricted expression and is exclusively observed in vertebrates [49]. Pacak–Zhuang syndrome is a syndrome resulting from somatic gain-of-function mutations in HIF2α encoded by the *EPAS1* gene, which occurs early in embryogenesis, and paraganglioma is one of the characteristics of Pacak–Zhang syndrome [55]. Based on *HIF2α* mutation, drugs can be used, which will be discussed in the following sections. Although both HIF1α and HIF2α interact with the same partner, HIF1β, and respond to similar elements, there might exist some selectivity in target gene activation between the two isoforms of HIFαs due to chromatin context-dependent regulation of gene expression in distinct cell types [47,49,56]. Both HIF isoforms can be stabilized and activated in cancer cells where they induce the expression of genes, such as *VEGFs* [57,58], which facilitates angiogenesis in solid tumors. Moreover, they directly or indirectly activate genes involved in cell proliferation, the epithelial-to-mesenchymal transition (EMT), apoptosis, metastasis, or tumor invasion [56]. Celeda et al. reported that while noncancerous human CB expresses HIF2α—a finding relevant for understanding its role in tumorigenesis—high levels of HIF2α accumulate specifically within the cells of human CB under physiological conditions; however, no such accumulation is observed for HIF1α [59].

Consequently, the HIF is a pivotal pathway supporting tumor growth by facilitating angiogenesis and promoting various tumor-associated phenotypes [47,48,49,60]. In CBTs, it has been suggested that the activation of HIFs stimulates carotid body growth, propels its progression, and regulates the expression of VEGFs, which will be discussed in the next section.

**Table 2:** Expression of HIFs in the carotid body.

Genes	Localization	Species	Detection Methods	Reference
*HIF1A*	Type I cells, Type II cells	Rats	Immunohistochemistry	Roux JC et al., 2005 [51]
*EPAS1*	Type I cells	Rats	Immunohistochemistry	Roux JC et al., 2005 [51]
	Carotid body	Human	Immunohistochemistry	Celada L et al., 2022 [59]

### 3.3. Vascular Endothelial Growth Factor (VEGF)

Over two decades ago, VEGFs were identified, isolated, and cloned as an essential factor in vasculogenesis and angiogenesis [61]. Although its primary target is endothelial cells, it has been shown to have multiple effects on other cell types [62]. VEGFs play a crucial role in maintaining vascular homeostasis across diverse tissues and cells; however, it also contributes to the molecular pathogenesis of tumor growth and metastasis. Increased VEGF expression is a characteristic feature of all VHL tumor types, and HIF dysregulation has been implicated in this phenomenon [63,64,65]. It has been proved that whether oxygen is plentiful or not, lacking VHL overproduces hypoxia-inducible mRNAs, including VEGF mRNA, and many hypoxia-inducible mRNAs, including the VEGFs mentioned above, are transcriptionally regulated by HIFs [66].

Moreover, studies have confirmed that under hypoxia conditions, HIF-1α is activated and regulates VEGFs and other transcription factors to participate in tumor new angiogenesis [67]. The mechanism may be that in hypoxia-driven angiogenesis, hypoxia activates the PI3K/AKT pathway, prevents post-translational hydroxylation of HIF-1α and subsequent degradation of HIF-1α, allowing it to accumulate, then transfer to the nucleus, and form a transcription initiation complex, initiating target gene transcription, leading to an increase in corresponding protein products, including enhanced expression of VEGFs [68]. Moreover, the PI3K pathway regulates the synthesis of VEGF proteins and the hypoxia-activated PI3K/Akt/mTOR pathway [69]. It is also reported that HIF2α can activate various genes encoding molecules, including VEGFs. When low oxygen levels are present, there is a loss of PHD activity, which limits VHL binding to HIF2α. Without VHL binding and marking for proteasomal degradation, HIF2α stabilizes, accumulates, and translocates into the nucleus [70]. Once in the nucleus, HIF2α heterodimerizes with HIF-1β and recruits p300/CBP co-activators to form an active HIF transcription complex [70]. The HIF transcription complex then binds to hypoxia response elements (HREs), resulting in up-regulated transcription of hypoxia-inducible genes such as VEGFs [70].

As CBTs is a kind of hypervascular tumor, VEGFs and its receptors are researched in CB cells of humans and rats (its expression in CB cells is shown in Table 3 [71,72,73,74,75,76,77,78]). Based on the above statements, researchers have become increasingly interested in its involvement in CBTs related to hypoxia.

In the carotid body (CB), type I cells have been demonstrated to express VEGFs, as well as its receptors Flt-1 (VEGFR1) and Flk-1 (VEGFR2) [79]. VEGFs exert their effects on Flk-1, regulating the hyperplasia of type I cells and promoting neovascularization through interaction with Fit-1 in endothelial cells [80]. Given that exposure to hypoxia is known to enhance CB microvascularization and the number and size of glomus cells [80], extensive research has focused on investigating the regulation of VEGF expression under this stimulus [74,78,81,82]. Extensive research has focused on regulating VEGF expression under this stimulus [74]. Additionally, HIFs can activate VEGF expression as well [57,58]. These findings underscore the crucial role played by VEGFs in hypoxic responses within CBTs.

### 3.4. Functions of Inflammatory Cytokines (ICs) in Carotid Body Concerning Hypoxia

Inflammatory factors have been shown to play a significant role in the physiology and plasticity of the carotid body (CB). Glomus cells produce proinflammatory cytokines, including interleukin (IL)-1β, IL-6, and tumor necrosis factors (TNFs), along with corresponding receptors that regulate CB excitability, catecholamine release, and chemoreceptor discharge [79,83]. Notably, inflammatory cytokines, such as IL-1α/β, IL-6, and TNFs, are expressed in type I cells of the rat CB [84]. Additionally, in situ hybridization has localized IL-6 expression in type II cells, while ELISA measurements have detected elevated IL-6 concentrations within the CB lysate (Table 4) [74,85,86,87].

Xue and his colleagues first investigated the effects of proinflammatory cytokines on CB neurogenesis [88]. Exposure to intermittent hypobaric hypoxia (IHH) promoted extracellular signal-regulated kinase (ERK) 1/2 phosphorylation, which determines neuronal progenitor cell fate, as well as the increased expression of tyrosine hydroxylase (TH) and nestin, a specific neuronal stem cell marker in rat CB. Additionally, the intraperitoneal administration of IL-1 had an additive effect on IHH. These results suggest that treatment with IL-1 may increase CB plasticity, while ERK1/2 appears to play a role in neurogenic signaling in CB [88].

The effect of exogenous cytokine administration on dissociated glomus cells was examined in CIH-exposed CB [74] and unstimulated chemoreceptor organs. A study conducted by Fan and his colleagues [89] investigated the impact of IL-6 on Ca^2+^ levels and catecholamine (CA) secretion in rat CB cell cultures. Following IL-6 administration, treated cells exhibited increased Ca^2+^ levels, as determined by fluorometric measurements. Furthermore, amperometric analysis revealed that IL-6 induced catecholamine release using glomus cells, abolishing this response by the calcium channel blocker Cd^2+^. These data confirm the carotid body (CB)’s ability to respond to proinflammatory cytokines, highlighting its role in sensing inflammation and transmitting this information to the brain [89]. The expression of proinflammatory cytokines using CB was also investigated in human samples obtained from surgical patients undergoing elective head and neck cancer surgery. CB slices exposed to sustained hypoxia for 1 h exhibited an increased release of IL-1 [84].

## 4. Treatment Based on Hypoxic Signaling Pathways in CBTs

The current treatment strategy for CBTs primarily focuses on active surveillance, external beam radiation, and surgery [90]. The hypoxic condition of carotid tissue is a common occurrence in CBTs and results in cellular changes that contribute to aggressive behavior and therapeutic resistance. While hypoxia induces resistance to various treatments, it mainly affects photon radiotherapy as it relies on generating free radicals for its cytotoxic effect [91]. PET imaging has demonstrated significant levels of hypoxia in a wide range of tumors, and detecting and modifying the hypoxic environment are crucial approaches in CNT treatment. Fluoromisonidazole (FMISO), a nitroimidazole compound, has been extensively studied and found to possess the most comprehensive experience among various hypoxia imaging agents [92]. Recently, Lu et al. [93] used reduced nanographene oxide (rNGO) sheets with MnO_2_ nanoparticles, doxorubicin, and methyl blue as photothermal agents to trigger further photodynamic therapy and chemotherapy. In their study, MnO_2_ acted as a catalyst for hydrogen peroxide and generated oxygen as an essential component for photodynamic therapy. This innovative approach opens up new possibilities for implementing multiple treatment strategies in CBTs.

As we discussed above, there are several hypoxic signaling pathways in CBTs, so some drugs based on these targets have been designed for treatment. Several treatment methods targeting VEGFs have been studied or applied in paraganglioma. One approach uses receptor tyrosine kinase inhibitors, such as sunitinib [94], which inhibit the signaling pathways involved in VEGF-mediated angiogenesis. Studies have shown that sunitinib can lead to tumor regression and improved progression-free survival in patients with progressive malignant paragangliomas [95,96]. Another approach is the inhibition of HIF2α, a transcription factor that regulates VEGF expression. Belzutifan, a specific inhibitor of HIF2α, disrupts its binding to its partner protein HIF1β and may show benefits in patients with paragangliomas caused by mutations in genes like EPAS1 [55,97]. It is important to note that these treatment methods targeting VEGFs are still being evaluated and may not be effective for all patients [98]. Further research and clinical trials are needed to determine their efficacy and safety in treating paragangliomas.

Tetraazacyclododecane tetraacetic acid octreotate (DOTATATE) is a radiopharmaceutical agent used to treat certain tumors, including paragangliomas and pheochromocytomas. It targets somatostatin receptors, specifically somatostatin receptor type 2 (SSTR2), often overexpressed in these tumors [26]. By binding to SSTR2, DOTATATE delivers a radioactive substance (usually lutetium-177 or yttrium-90) directly to the tumor cells, causing localized radiation therapy. The high affinity of DOTATATE for SSTR2 allows targeted radiation to be delivered to tumor cells while minimizing damage to surrounding healthy tissues. This makes it an effective treatment option for patients with metastatic or inoperable paragangliomas and pheochromocytomas. In addition to its therapeutic role, DOTATATE can also be used for diagnostic purposes [26]. It is commonly used in somatostatin receptor imaging, known as somatostatin receptor scintigraphy, to detect and localize tumors that express SSTR2 [26]. Overall, DOTATATE plays a crucial role in managing paragangliomas and pheochromocytomas by providing targeted radiation therapy and aiding in tumor detection and localization.

## 5. Future Expectations of CBTs

The pathogenesis of CBTs remains elusive despite the wide acceptance of pseudohypoxia as a primary factor. However, it is worth noting that not all CBT patients exhibit SDH mutations or reside in high-altitude regions. Other pathogenic factors, such as kinase signaling and Wnt-altered clusters, are also reported to be associated with paragangliomas. The mechanism of the two hypotheses in paraganglioma and CBTs still needs exploration and may have a vital function in the treatment of CBTs. Molecular diagnosis may be a future topic of CBT research. We can find new markers through omics studies, and based on these markers, more molecular functions can be explored. CBTs may be divided into different phenotypes and benefit treatment based on distinctive markers. Also, new materials and drugs for CBTs may be a focus issue for CBTs have different types. Examples of this, such as Belzutifan, an inhibitor of HIF2α, were discussed in the main text. We firmly believe that more markers like SDH and HIFs will be found in the future and play crucial roles in CBTs. Therefore, further investigations should be directed toward elucidating alternative etiological factors and developing corresponding therapeutic strategies.

## 6. Conclusions

The specific metabolic pathways underlying CBTs are not yet fully understood; however, there has been increasing focus on investigating hypoxic signaling pathways as a possible explanation for their high prevalence among individuals living at higher altitudes. Despite more than twenty years of research on hypoxic signaling, there remains a lack of comprehensive understanding regarding these signaling and markers. This review aims to clarify the functions associated with SDH, HIFs, VEGFs, and inflammatory cytokines related to hypoxic signaling in CBTs while exploring their potential roles in its development.

SDH is the most significant factor in hypoxic signaling pathways. SDH and FH mutations could lead to the persistence of HIFα in normal oxygen conditions, and SDH mutation could also signal PHDs via increasing ROS. Then, HIF subunits undergo degradation in the proteasome under normoxic conditions through a mechanism that involves active PHD enzymes and the subsequent interaction of HIFs with VHL protein, though a VHL mutation can interrupt this process and result in pseudohypoxia. The HIF is a core of hypoxic signaling pathways in CBTs, and HIFs can activate VEGF expression. Up-regulated VEGFs and hypoxia conditions can promote the neovascularization and hyperplasia of the CB. The hypoxia conditions also induce the expression of ICs to stimulate CB hyperplasia, ultimately leading to CBTs. Detecting hypoxic areas in tumors and improving the hypoxic area could enhance photon radiotherapy efficacy. In conclusion, markers related to hypoxia have substantial implications for CBT research; however, further exploration is still warranted.

## Figures and Tables

**Table 1:** Mutations of SDHx in the carotid body.

Gene	Chromosome	Frequency *	Proportion of Attributed Hereditary Paraganglioma	Inheritance Pattern
*SDHA*	5p15	<1%	0.6–3%	Autosomal dominant
*SDHB*	1p36.1	5%	22–38% 12–20% CBT	Autosomal dominant
*SDHC*	1q21	1%	4–8%	Autosomal dominant
*SDHD*	11q23	5%	30% 40–50% CBT	Autosomal dominant paternal inheritance
*SDHAF2*	11q13.1	<1%	Unknown	Autosomal dominant paternal inheritance

Note: Summarized from Gene Reviews [43] and Galan et al. [44]. * represents mutation frequency in paragangliomas.

**Table 3:** The expression of VEGFs in the carotid body.

Genes	Localization	Species	Detection Methods	Reference
*VEGF*	Type I cells	Rat	Immunohistochemistry	Lam et al., 2008 [71]
	Type I cells	Rat	Immunohistochemistry	Chen et al., 2003 [72]
	Carotid body	Rat	Immunohistochemistry	Di Giulio et al., 2009 [73]
	Carotid body	Rabbit	ELISA	Feng et al., 2008 [74]
	Type I cells	Rat	Double immunofluorescence	Belzunegui et al., 2008 [75]
	Type I cells	Rat	Immunohistochemistry	Felix et al., 2012 [76]
	Carotid body	Human	Immunohistochemistry	Zara et al., 2013 [77]
	Carotid body	Rat	qRT-PCR	Salman et al., 2017 [78]
*Flk-1*	Type I cells	Rat	Immunohistochemistry	Chen et al., 2003 [72]

**Table 4:** The expression of inflammatory cytokines in the carotid body.

Genes	Localization	Species	Detection Methods	Reference
*IL1B*	Type I cells	Rat	Immunohistochemistry	Del Rio et al., 2012 [87]
	Carotid body	Human	ELISA	Kåhlin et al., 2014 [86]
*IL6*	Type I cells	Rat	Immunohistochemistry	Del Rio et al., 2012 [87]
	Carotid body	Human	ELISA	Kåhlin et al., 2014 [86]
	Carotid body	Human	ELISA	Kåhlin et al., 2014 [86]
*TNFA*	Type I cells	Rat	Immunohistochemistry	Del Rio et al., 2012 [87]

## Data Availability

All data generated or analyzed during this study are included in this published article.
